# Improving strawberry plant resilience to salinity and alkalinity through the use of diverse spectra of supplemental lighting

**DOI:** 10.1186/s12870-024-04984-y

**Published:** 2024-04-08

**Authors:** Mohammad Reza Malekzadeh, Hamid Reza Roosta, Majid Esmaeilizadeh, Piotr Dabrowski, Hazem M. Kalaji

**Affiliations:** 1 Department of Horticultural Sciences, Faculty of Agriculture, Vali-e-Asr University of Rafsanjan, 7718817111 Kerman, Iran; 2 Department of Horticultural Sciences, Faculty of Agriculture and Natural Resources, Arak University, Arak, 38156-8-8349 Iran; 3 Department of Environmental Management, Institute of Environmental Engineering, Warsaw University of Life Sciences-SGGW, Nowoursynowska str. 159, Warsaw, 02-776 Poland; 4 Department of Plant Physiology, Institute of Biology, Warsaw University of Life Science, 159 Nowoursynowska St, Warsaw, 02-776 Poland

**Keywords:** Abiotic stresses, Chlorophyll fluorescence, LED, Photosynthesis

## Abstract

**Background:**

This study explores the impact of various light spectra on the photosynthetic performance of strawberry plants subjected to salinity, alkalinity, and combined salinity/alkalinity stress. We employed supplemental lighting through Light-emitting Diodes (LEDs) with specific wavelengths: monochromatic blue (460 nm), monochromatic red (660 nm), dichromatic blue/red (1:3 ratio), and white/yellow (400–700 nm), all at an intensity of 200 µmol m^-2^ S^-1^. Additionally, a control group (ambient light) without LED treatment was included in the study. The tested experimental variants were: optimal growth conditions (control), alkalinity (40 mM NaHCO_3_), salinity (80 mM NaCl), and a combination of salinity/alkalinity.

**Results:**

The results revealed a notable decrease in photosynthetic efficiency under both salinity and alkalinity stresses, especially when these stresses were combined, in comparison to the no-stress condition. However, the application of supplemental lighting, particularly with the red and blue/red spectra, mitigated the adverse effects of stress. The imposed stress conditions had a detrimental impact on both gas exchange parameters and photosynthetic efficiency of the plants. In contrast, treatments involving blue, red, and blue/red light exhibited a beneficial effect on photosynthetic efficiency compared to other lighting conditions. Further analysis of JIP-test parameters confirmed that these specific light treatments significantly ameliorated the stress impacts.

**Conclusions:**

In summary, the utilization of blue, red, and blue/red light spectra has the potential to enhance plant resilience in the face of salinity and alkalinity stresses. This discovery presents a promising strategy for cultivating plants in anticipation of future challenging environmental conditions.

**Supplementary Information:**

The online version contains supplementary material available at 10.1186/s12870-024-04984-y.

## Background

Salinity/alkalinity stress is a major abiotic stress factor that can hinder the natural growth and development of plants, leading to significant damage to agricultural production and land productivity. Saline soils are predominantly composed of mixed salts such as NaCl, Na_2_SO_4_, Na_2_CO_3_, and NaHCO_3_. Neutral salts like NaCl and Na_2_SO_4_ cause salinity stress, with a pH value ranging from 7 to 8. Alkaline stress, on the other hand, is caused by alkaline salts like NaHCO_3_ and Na_2_CO_3_, which have a high pH value exceeding 8.5. Some soils may also experience mixed stress. Salinity/alkalinity stress not only damages the plant cell structure, cell osmotic pressure stability, and nutrition mechanism but also results in an ecological environment with limited plant species, low biodiversity, and rough soil conditions [1–3]. Mixed salinity and alkalinity stress often occurs naturally, and the effect of mixed salinity and alkalinity stress is very different from individual salt or alkali stresses due to the significant interaction between ion toxicity, osmotic effects, and high pH. Osmotic effects and ionic toxicity effects depend on salt concentration, while pH effects depend on buffer capacity [4]. This means that the higher the concentration of salt and alkalinity, the greater the buffer capacity. Therefore, it is more difficult for plants to adapt. This interaction can lead to severe plant damage. Similar effects of mixed salt and alkali stress have been reported in sunflower [5].

Photosynthesis in plants is a complex process that involves the absorption of light and its conversion into chemical energy. Chlorophyll fluorescence is a simple and effective way to measure how plants respond to stressful conditions [6]. This technique enables the assessment of a plant’s status in unfavorable environmental situations by analyzing fluorescence data. The parameters measured are closely linked to the performance of the photosystem II. When a green plant is exposed to stress, which affects photosynthetic metabolism directly or indirectly, the function of chlorophyll fluorescence changes. Since the variable fluorescence of chlorophyll strongly reacts to changes in the activity of photosystem II, fluorescence quenching analysis provides information on energy absorption, utilization, and dissipation, as well as electron transport in photosystem II [7]. Fluorescence reflectance represents the photochemical efficiency of the photosynthetic apparatus and offers useful data on the functional and structural characteristics of the mechanisms engaged in photosynthetic electron transport [8].

The architecture of the leaves is determined by the size and shape of the cells, as well as the number of cells per unit area. This optimal structure allows light to pass through and facilitates the emission of carbon dioxide from the leaf space to the chloroplasts, where photosynthetic reactions take place. Chlorophyll and carotenoids are photosynthetic pigments responsible for light absorption and photochemical reactions in chloroplasts [9]. When growing plants in greenhouses, additional light is often needed to supplement natural sunlight, which is generally less intense in winter than in summer. Additionally, the length of the day is shorter in winter. High-pressure sodium lamps were traditionally used in greenhouses to enable winter production, but LEDs have replaced them due to producing less heat and being more energy-efficient [10, 11]. Moreover, the wavelengths of LEDs can be adjusted according to the growing needs of each crop. LEDs offer greenhouse growers sustainable and widespread production opportunities, including off-season production [12, 13]. Several studies have shown that a combination of red, blue, and white LEDs can increase photosynthetic pigments as well as the net rate of photosynthesis in cherry tomatoes [14], leaf area [15], and the size of strawberry fruit [16]. It has been shown that light spectrums, especially red and blue/red light, have caused a significant increase in the level of phenol and antioxidants in *Thymus vulgaris* L [17]. This shows that the light spectra can also play a role in increasing the tolerance of plants to stresses by increasing the antioxidant properties.

The term “light quality” refers to the combination of light spectra that impact the growth and development of plants, with effective wavelengths crucial for photosynthesis [18]. Photosynthesis serves as the foundation for plant growth, influencing factors such as leaf growth, photosynthetic carbon absorption, and regulation of stomatal movement, chloroplast structure, and accumulation of photosynthetic pigments. Different light spectra exert varying effects on plants, depending on the plant type, organ, and tissue. For instance, blue and red light can induce stomatal opening, while green light may cause stomatal closure. Blue light aids in chloroplast growth, whereas a combination of red, blue, and green lights can increase leaf area. Red light, in particular, enhances the accumulation of photosynthetic products. Higher plants and green algae predominantly perform photosynthesis in the orange and red-light spectrum, with blue and violet light resulting in lower amounts of photosynthesis, and green light the least. Ultraviolet light can reduce the electron transport activity of photosystem II [19].

Plants possess unique photoreceptors that absorb specific wavelengths of incoming light. These include phytochromes that absorb red and far-red light, cryptochromes, and phototropins [20]. These photoreceptors coordinate growth and photosynthetic functions in response to changes in the environment. Additionally, red- and far-red light-sensitive phytochromes and blue-light-sensitive cryptochromes play crucial roles in modulating photomorphogenesis by coordinating large-scale changes in gene expression [21].

In greenhouses, especially during winter and rainy days in summer, natural light may be insufficient for optimal plant growth and development, resulting in extended cultivation periods and delays in fruit production. Hence, the use of additional artificial light spectra can provide a solution to this problem. Therefore, examining the impact of supplementary light quality on plant growth can be a practical approach to enhance plant productivity [22]. LED lamps, being low-power-consuming, compact, easy to transport, and long-lasting, are ideal for horticultural lighting in both small- and large-scale operations [23].

It has been suggested that different photoreceptors play a role in the interrelationship between light signaling pathways and stress response, regulating responses to both biotic and abiotic stresses. To survive and prepare for future stress, plants have evolved a complex relationship between light signaling and various stress response pathways [24]. Another study demonstrated that four wavelengths of blue, red, green, and white LEDs significantly affect the plant’s antioxidant defense system, indicating the potential of light spectrum manipulation as a strategy under stress conditions [25].

**Fig. 1:**
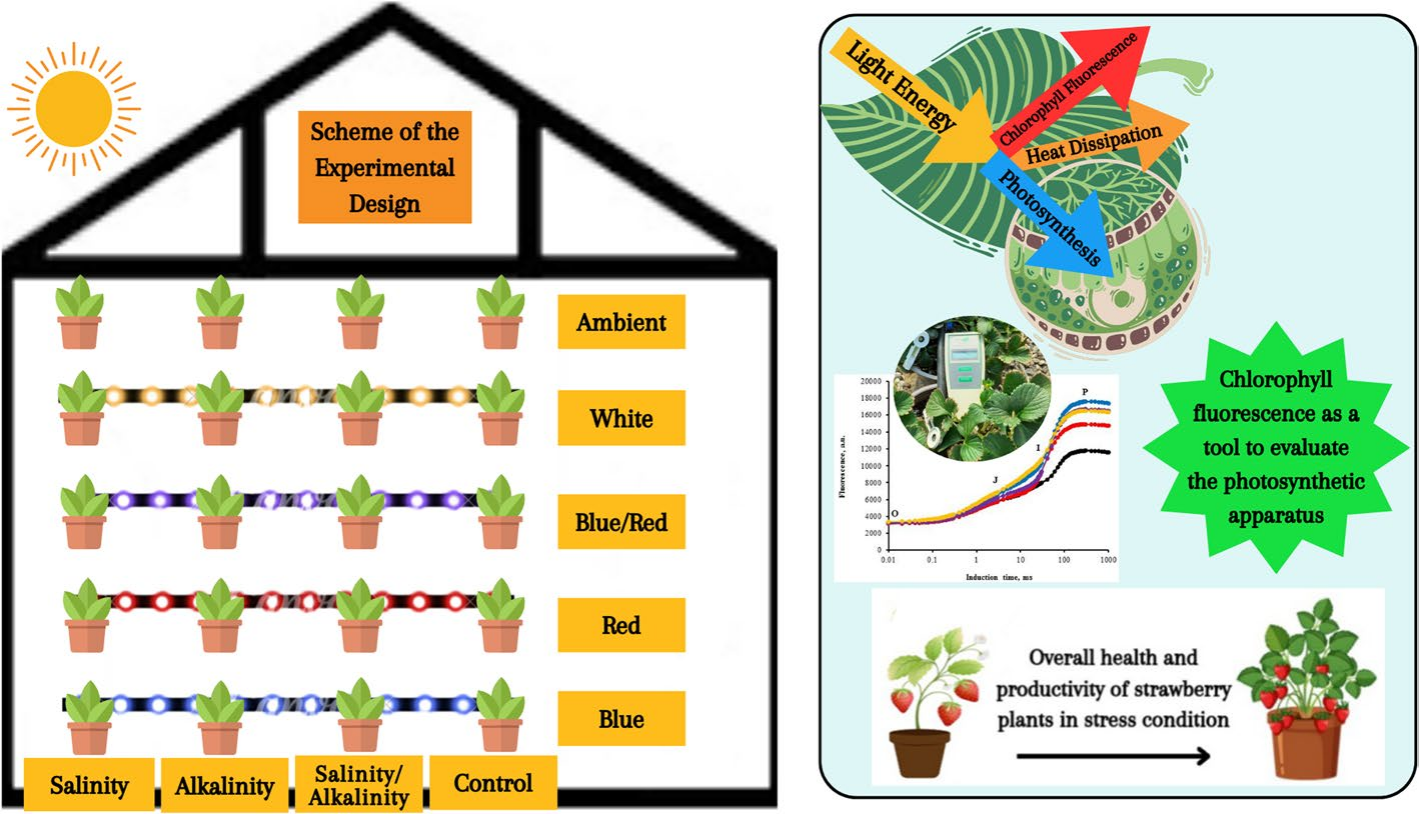
Schematic figure of the experimental design and the effectiveness of the chlorophyll fluorescence technique in evaluating the photosynthetic apparatus

In our previous experiments, the separate effects of salinity and alkalinity stress were investigated. However, since both stresses may coexist naturally in the soil, this experiment aimed to explore the effect of salinity/alkalinity stress on the Sabrina variety, a commercial strawberry variety. Specifically, we sought to determine whether different complementary light spectra could enhance the tolerance of strawberry plants under salinity/alkalinity stress conditions. In addition to studying the separate effects of salinity and alkalinity stresses, we investigated their combined effects. Furthermore, we examined how complementary light spectra affect parameters such as plant gas exchange and the electron transport chain of the photosynthetic apparatus in strawberry plants under stress conditions. We hypothesized that the use of additional lighting would positively impact the functioning of the photosynthetic apparatus of strawberry plants, thereby reducing the adverse effects of salinity/alkalinity stress. Figure 1 illustrates the schematic diagram of the experimental design and the technique of chlorophyll fluorescence used to evaluate the photosynthetic apparatus.

## Materials and methods

### Plant material, planting conditions, and light treatments

The experiment was conducted at Vali-e-Asr University of Rafsanjan, Iran, in a research greenhouse located at Latitude 30° 21’ 17.6004’’ N and Longitude 56° 0’ 9.738’’ E, at an elevation of 1545.924. Strawberry bare root plants (*Fragaria × ananassa* Duch, cv. Sabrina) were obtained from a nursery in Kurdistan, Iran. The plants were grown in a 4-liter pot containing a mixture of cocopeat and perlite in a 70:30 ratio. Each pot had two plants, and three pots were used as experimental units. The plants were cultivated in a greenhouse with a temperature of 25/15 ± 2 ºC (day/night), a photoperiod of 11.13 h (light/dark), and relative humidity of 50 ± 10%. During the growth period, the plants were fertigated with the Morgan nutrient solution [26], which had an Electrical Conductivity (EC) of 1.4 dS .m^-^1 and a pH of 6.5.

**Fig. 2:**
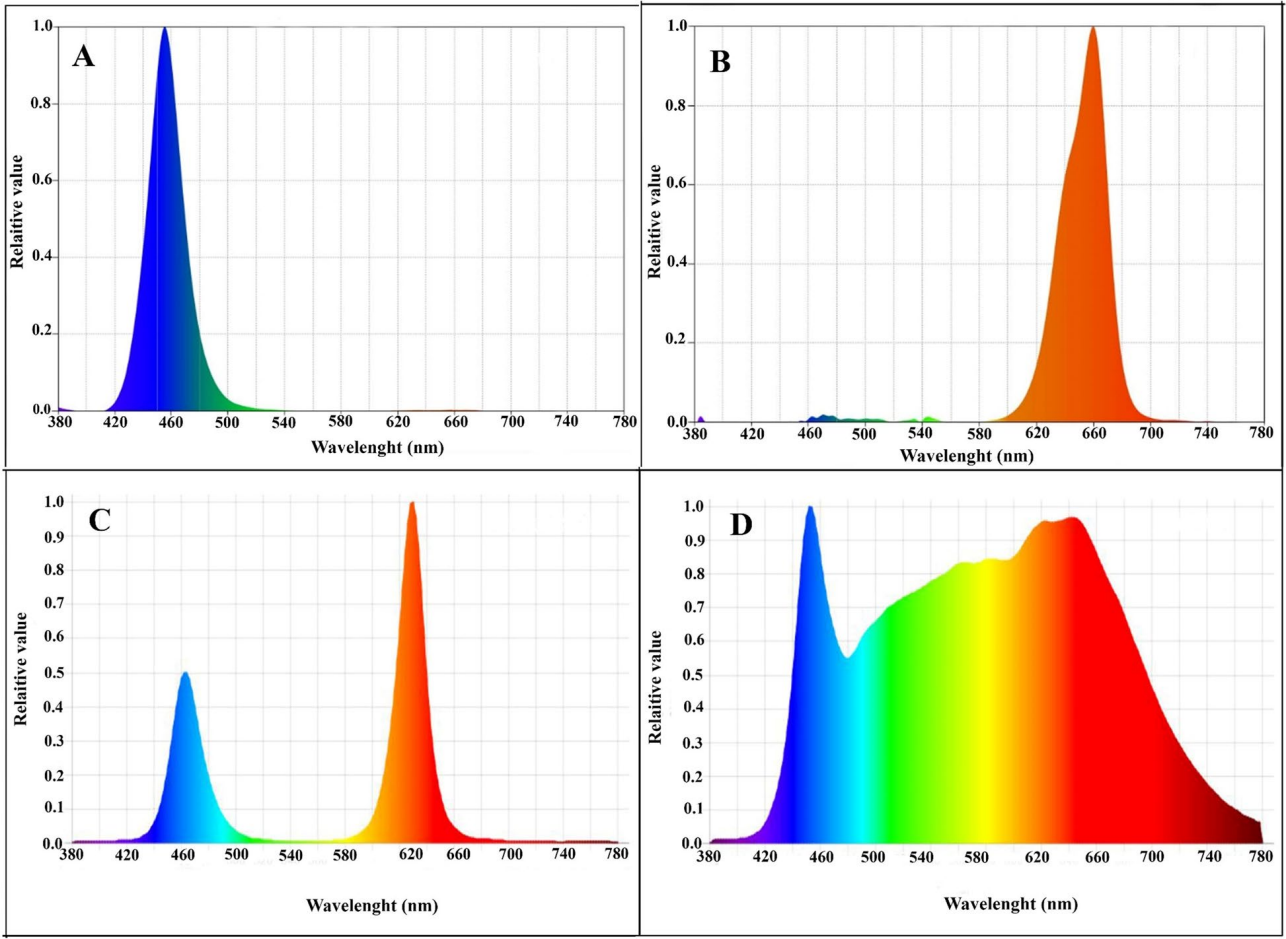
Spectral composition of different spectral LEDs **A** monochromatic blue; **B** monochromatic red; **C** dichromatic blue/red (1:3); **D** dichromatic white used during the experiment

The plants were grown under LED lights of different spectral ranges provided by Parto Roshd Novin Company, Iran growlight. The spectral ranges included monochromatic blue (with a peak at 460 nm), monochromatic red (with a peak at 660 nm), dichromatic blue/red (at a ratio of 1:3), white (covering the range of 400 to 700 nm) as well as a control group with no LED treatment (Fig. 2). The light intensity was set at 200 µmol m^−2^ s^−1^ for an 11-hour light period. The plants were exposed to four different stress levels, including control, salinity (80 mM NaCl), alkalinity (40 mM NaHCO_3_), and salinity/alkalinity (S/A). The salinity and alkalinity treatments were applied twenty days after planting, and each pot received 100 ml of NaHCO_3_ and NaCl to ensure uniform stress levels for the plants. To determine the salinity and alkalinity concentrations, we started the treatment of salinity and alkalinity from lower concentrations and gradually increased it until we observed the effects of stress in plants at a concentration of 40 mM NaHCO3 and 80 mM NaCl and we chose these concentrations to investigate the effects of complementary light spectrums on reducing the effects of these stresses. The stresses were continued until the end of the experiment and data collection. The length of the experiment period was 80 days, and the application of stress treatments started on the 20th day and continued for 60 days (until the end of the test). Photosynthetic parameters were measured 40 days after stress treatments were applied when the plant was sufficiently mature and had enough mature leaves.

### Photosynthetic pigments and carotenoids

To determine the total chlorophyll (TChl), chlorophyll *a* (Chl *a*), and chlorophyll *b* (Chl *b*), as well as carotenoid (Car) contents, three fully extended leaves were collected from each pot. 0.25 g of fresh leaves were ground with 10 ml of 80% aqueous acetone in a cold mortar. The resulting extract was filtered and centrifuged, and the absorbance was measured at 470, 646, and 663 nm using a spectrophotometer-U-2000 (Hitachi Instruments, Tokyo, Japan). The TChl, Chl *a*, Chl *b*, and carotenoid were calculated and expressed as mg.g^-1^FM [27].

### Leaf gas exchange measurements

The parameters of gas exchange in plants were measured using a portable photosynthesis system (ADC BioScientific Ltd, LCpro-SD, Hoddesdon, UK) 60 days after planting. Measurements were made on a sunny day between 10:00 AM and 2:00 PM. The leaves were placed in the cuvette for 3 min, and then the measurement was done. The parameters measured include net CO_2_ assimilation rate (*PN*, µmol CO_2_ m^-2^.s^-1^), stomatal conductance (*gs*, mol H_2_O m^-2^.s^-1^), transpiration (*E*, mmol H_2_O m^-2^.s^-1^), Sub-stomatal CO_2_ concentration (*C*_*i*_, µmol CO_2_ mol^-1^), instantaneous carboxylation efficiency (*A/C*_*i*_), water use efficiency (*WUE*, µmol CO_2_ .mol^−1^ H_2_O), and intrinsic water use efficiency (*WUEi*, µmol CO_2_ .mol^−1^ H_2_O). The measurements were taken on fully expanded leaves at around 10:00 AM and 2:00 PM. The air temperature and light intensity in cuvette during measurements were ambient. The CO_2_ concentration was 400 ppm and molar flow of air was 200 mol s^-1^ [28].

### Chlorophyll fluorescence measurements

To measure the photosynthetic efficiency, a portable fluorometer PEA (Hansatech Inc. Co., UK) was used to evaluate chlorophyll fluorescence 60 days after planting. Fully mature leaves of each pot were adapted to a dark time for 15 min by fixing special tags on each leaf before measurement. Then, the sensor cup was mounted on the leaf for calculation. The chlorophyll a fluorescence transient was caused by a saturating photon flux density of 3.500 µmol (photon) m^−2^ s^−1^. This was given by three light-emitting diodes (peak 650 nm) to produce fluorescence curves ranging from F_0_ to F_m_. For all treatments, the fluorescence curves were measured at different times, i.e., F_t_ (fluorescence at time “t” after the onset of actinic illumination), F_0_ (minimum fluorescence intensity), F_j_ (fluorescence intensity at the J-step), F_I_ (fluorescence intensity at the I-step), and F_p_ (maximum fluorescence intensity at peak P of OJIP). The PSII parameters obtained from the OJIP transient were evaluated using the Strasser et al. (2010) methods. To investigate the effects of stress and complementary light spectra on the fast fluorescence curve, a relative variable fluorescence curve was drawn. The V_t_ value, calculated as (F_t_ – F_0_) / (F_m_ – F_0_), was used. Changes in OJIP fluorescence were determined by subtracting the fluorescence (O-P) values recorded in stressed plants from those recorded in plants control [7]. Parameters for chlorophyll fluorescence are listed in Additional file 1.

### Experimental design and data analysis

The experiment was factorial in the form of a completely randomized basic design with two factors included five different light spectrum levels (blue, red, blue/red, white and no LED treatment) and four different stress levels, including control, salinity, alkalinity, and salinity/alkalinity (S/A) in three replications and three single plants per pot. The data collected was analyzed using SAS software version 9.4. Parameters were statistically analyzed by the two-way ANOVA model, where the first factor was the level of light spectrum and the second was the stress. When the variance analysis (ANOVA) indicated significant treatment effects, significant mean variations (*P* < 0.01) were calculated using the LSD Multiple Range Test. Biophysical parameters were measured using PEA Plus software version 1.12 (http://www.hansatech-instruments.com). Pearson’s correlation coefficient was used to determine the relationship between the studied parameters. Origin Pro software version 2023b was used to draw correlation diagrams (https://www.originlab.com/2023). The biplots were made using Excel software version 2016. XLSTAT software version 2015.5.01.22537 was used for principal components analysis.

## Results

### Photosynthetic pigments

**Fig. 3:**
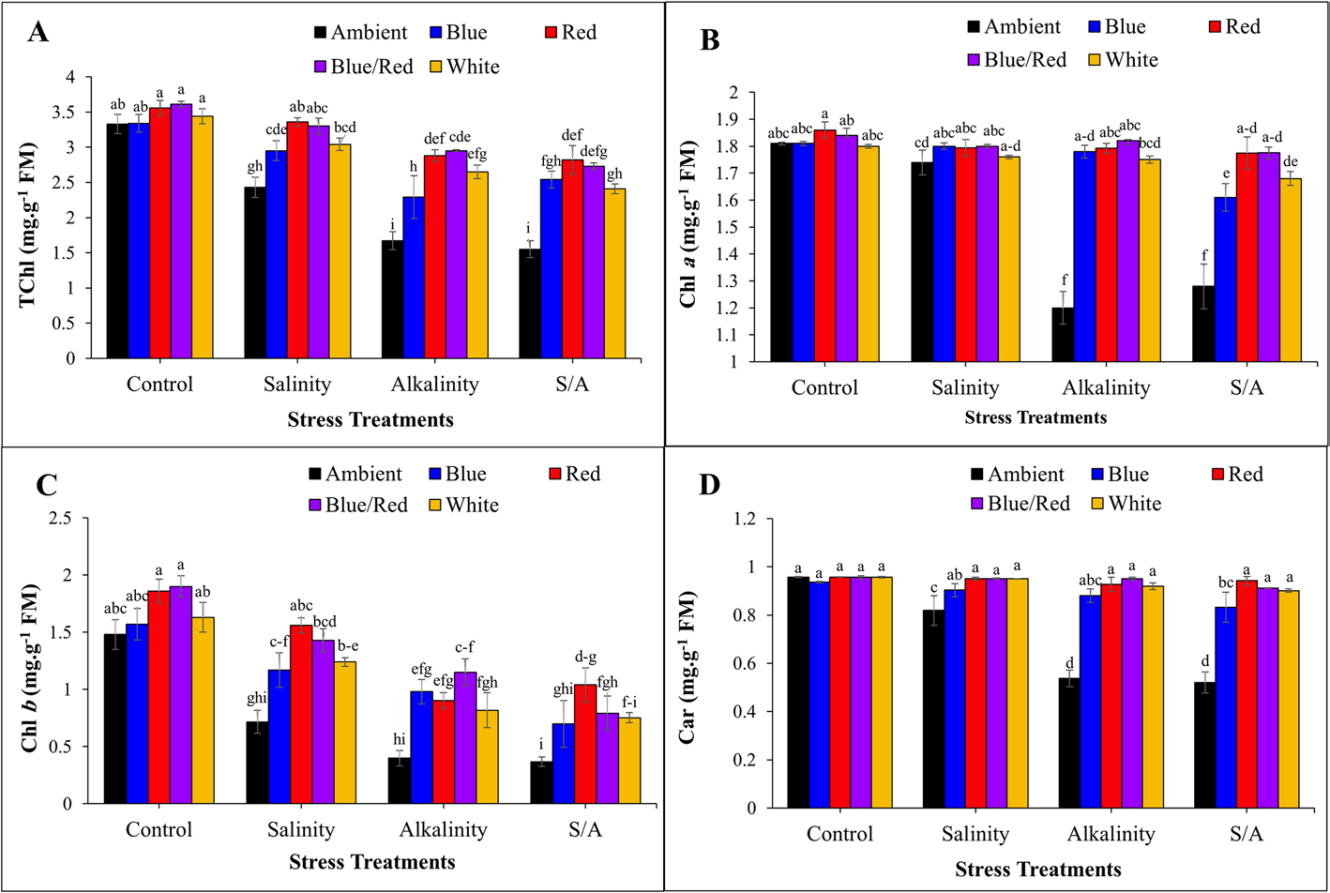
Changes in **A** total chlorophyll; **B** chlorophyll a; **C** chlorophyll b; **D** carotenoids under complementary light spectrums and stress condition in strawberry cv. Sabrina. Bars with different letters show significant differences at *P* ≤ 0.05 (LSD). Vertical bars indicate the standard errors of three replicates

 The results showed that in the control conditions, supplementary light treatments had no significant effect on total chlorophyll. In all stress conditions, supplemental light treatments significantly increased total chlorophyll compared to the ambient light treatment, so that the lowest total chlorophyll in all stress conditions was recorded in the ambient light treatment (Fig. 3A). In control, salinity, and alkalinity treatments, supplementary light treatments did not show significant difference on chlorophyll *a* levels. However, under alkalinity conditions and combined salinity/alkalinity stress, light treatments significantly increased chlorophyll *a* levels compared to the ambient light (Fig. 3B). The light treatments had no significant difference with each other on chlorophyll *b*. In the conditions of salinity and alkalinity stress, supplementary light treatments caused a significant increase in chlorophyll *b* compared to the ambient light. The lowest amount of chlorophyll *b* was recorded under the conditions of salinity/alkalinity stress and ambient light (Fig. 3C). Supplemental light treatments significantly increased carotenoid levels in strawberry plants under salinity and alkalinity stress conditions compared to the ambient light. Under salinity/alkalinity stress conditions, red, blue/red, and white supplementary light treatments significantly increased carotenoid levels compared to the other light treatments (Fig. 3D).

### Leaf gas exchange analyses

**Fig. 4:**
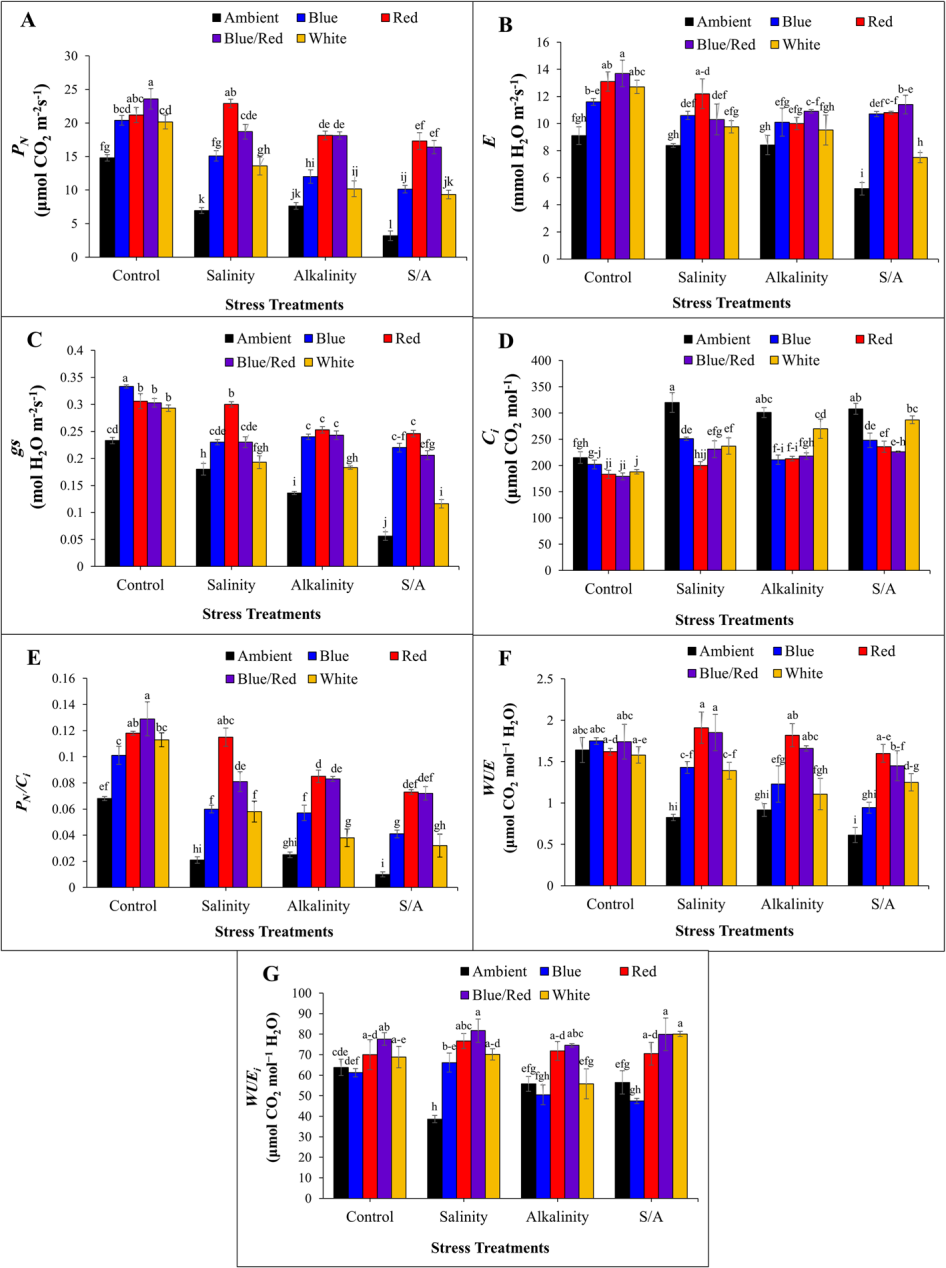
Changes in **A **net CO_2_ assimilation rate (*P*_*N*_); **B** transpiration rate (*E*); **C** stomatal conductance (*g*_*s*_); **D** Sub-stomatal CO_2_ concentration (*C*_*i*_); **E** instantaneous carboxylation efficiency (*P*_*N*_*/C*_*i*_); F: water use efficiency (*WUE*); **G** intrinsic water use efficiency (*WUE*_*i*_) under the effect of complementary light spectrums and stress condition in strawberry cv. Sabrina. Bars with different letters show significant differences at *P* ≤ 0.05 (LSD). Vertical bars indicate the standard errors of three replicates

 The results showed that the stress condition reduces the net CO_2_ assimilation rate (*PN*). Red and blue/red spectrum significantly increased *PN* in plants grown under salinity, alkalinity, and salinity/alkalinity stress conditions compared to other light treatments (Fig. 4A). The stress conditions also caused a decrease in the transpiration rate (*E*) and stomatal conductance (*gs*) when compared to the plants without any stress treatment.It was observed that under salt stress, red complementary light spectrum significantly increased gs in strawberry plants. In the case of alkalinity stress condition, it was observed that the blue, red, and blue/red light spectrum caused a significant increase in *gs*. When plants were subjected to salinity/alkalinity stress, the complementary blue and red light spectrum, along with their combination, had a significant effect on the *E* and *gs* compared to the other light treatments (Fig. 4B, C). Supplemental blue, red, and their combination light treatments significantly reduced substomatal CO_2_ concentration (*Ci*) under salinity, alkalinity, and salinity/alkalinity stress conditions compared to the ambient light treatment (Fig. 4D). It was also observed that complementary red light under salinity stress conditions and red and blue/red light under alkalinity and salinity/alkalinity stress conditions significantly increased instantaneous carboxylation efficiency (*P*_*N*_*/C*_*i*_) when compared to other complementary light spectrums (Fig. 4E). In the control condition, the complementary light treatments did not have a significant difference with each other on the *WUE* parameter. Complementary red and blue/red light treatments under salinity and alkalinity stress conditions significantly increased *WUE* in strawberry plants (Fig. 4F). Under alkalinity stress conditions, red and blue/red complementary light spectrum treatments significantly increased the *WUE*_*i*_ compared to the other light spectrums. It was observed that plants grown under salinity and salinity/alkaline stress conditions, red, blue/red, and white complementary light significantly increased *WUE*_*i*_ compared to the other complementary light spectrums and did not different significantly from each other (Fig. 4G).

### Prompt chlorophyll a fluorescence

**Fig. 5:**
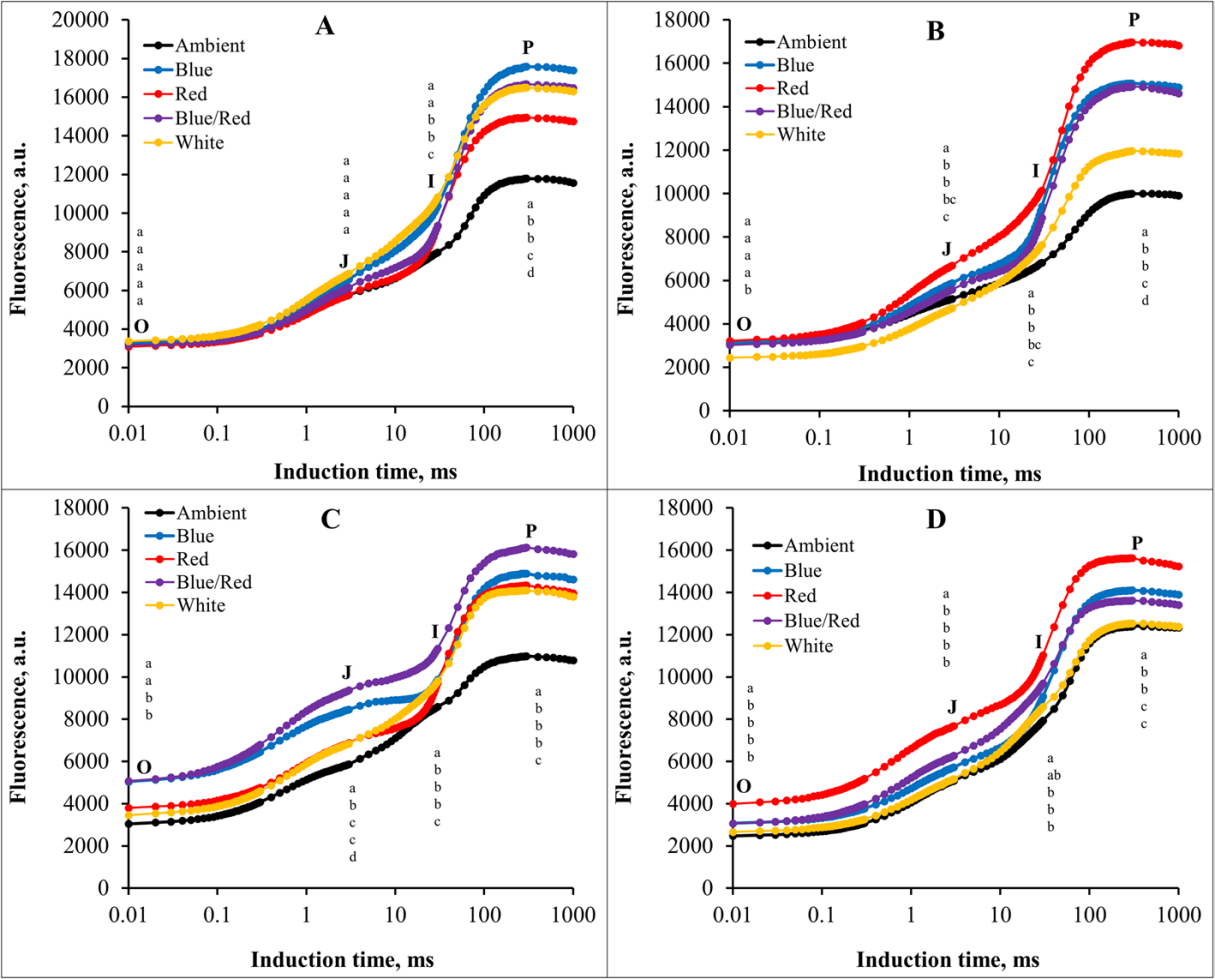
The chlorophyll a fluorescence induction curves for strawberry cv.Sabrina under various stress conditions: **A** control,**B** salinity stress, **C** alkalinity stress, and **D** combined salinity and alkalinity stress. Means within one point marked by one letter are not differ significantly. These stressors were applied in different light spectra, including blue, red, blue/red, white, and ambient light. The biophysical parameters were assessed using “PEA Plus” software version 1.12, available at http://www.hansatech-instruments.com. Graphs were drawn using Microsoft Excel version 2016, accessible at https://www.microsoft.com

The study found that using supplementary light in different stress conditions resulted in changes in the chlorophyll fluorescence induction curve, compared to the conditions without supplementary light. The results showed that the induction curve of chlorophyll fluorescence decreased significantly under stress conditions compared to control conditions. In the absence of stress conditions, blue light was the most effective at P step. Additionally, red and blue/red light were able to reduce the F_0_ point more than other light treatments (Fig. 5A). Red light had the most significant impact on increasing the F_m_ point under salinity stress conditions, while white light had a lower F_0_ point than other light treatments (Fig. 5B). In alkalinity stress conditions, blue/red supplementary light increased the induction curve of chlorophyll fluorescence in J, I, and P points (Fig. 5C). Under salinity/alkalinity stress conditions, red light increased the chlorophyll fluorescence curve at all points, while ambient light and white supplementary light treatments were less effective than other light treatments. The ambient light treatment had a lower level of F_m_ point in all stress treatments (Fig. 5D).

### Chlorophyll fluorescence parameters and induction curves

**Fig. 6:**
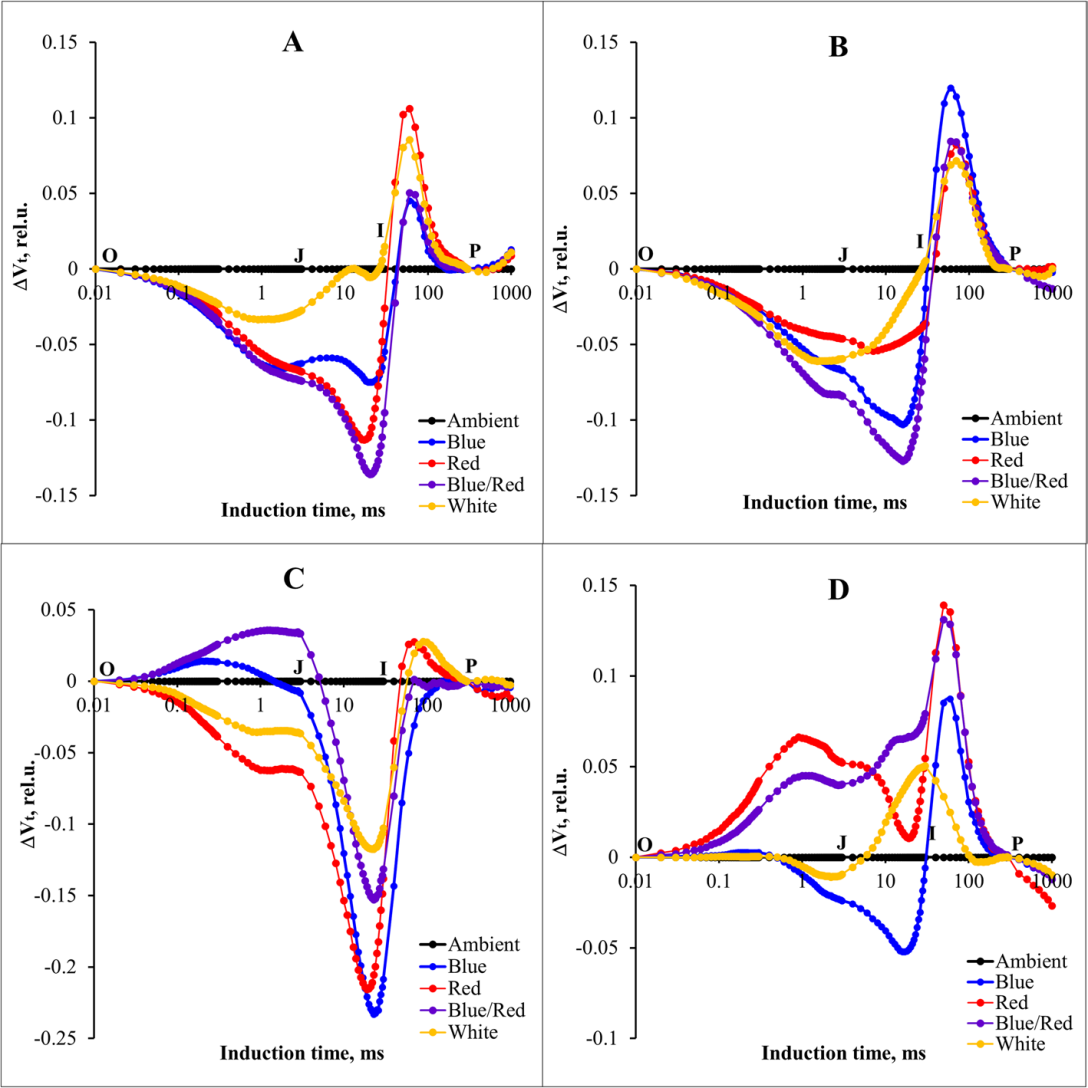
Differential curves of ΔVt of chlorophyll a fluorescence in strawberry cv.Sabrina under various stress conditions: **A** control, **B** salinity, **C** alkalinity, and **D** combined salinity and alkalinity stress. These stressors were applied across different light spectra, including blue, red, blue/red, white, and ambient light. The biophysical parameters were evaluated using “PEA Plus” software version 1.12, accessible at http://www.hansatech-instruments.com. Graphs were drawn using Microsoft Excel version 2016, which can be found at https://www.microsoft.com

The study found that different supplementary light treatments in various stress conditions significantly influenced the fast fluorescence of strawberry plants in J (V_J_) and I (V_I_) stages. The results suggest that different complementary light spectra in varying conditions of salinity, alkalinity, and their combination have significant effects on different phases of the curve. The positive effect in plants under without-stress conditions and other stress conditions at points J and I was caused by the effect of complementary blue, red, and their combination (Fig. 6A-D).

**Fig. 7:**
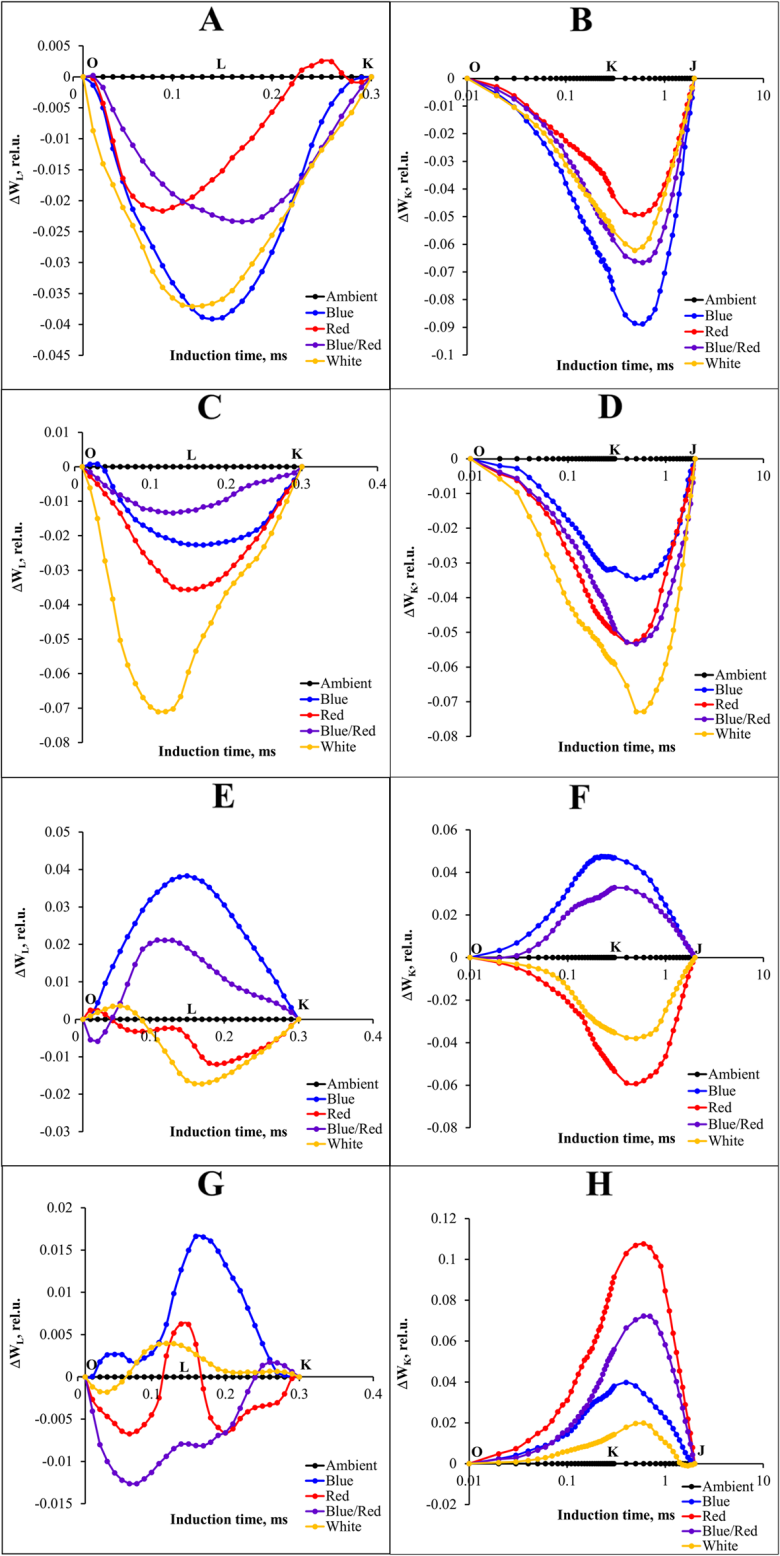
Differential curves of ΔW_K_ and ΔW_L_ of chlorophyll a fluorescence in strawberry cv.Sabrina under various stress conditions. **A**, **B** control; **C**, **D** salinity; **E**, **F** alkalinity; **G**, **H** combined salinity and alkalinity stress. These stressors were applied across different light spectra, including blue, red, blue/red, white, and ambient light. The biophysical parameters were evaluated using “PEA Plus” software version 1.12, accessible at http://www.hansatech-instruments.com. Graphs were drawn using Microsoft Excel version 2016, which can be found at https://www.microsoft.com

To accurately evaluate the effects of stress and complementary light spectra on fluorescence changes, differential curves were drawn for the L and K bands occurring between the O and J points. The curves for these bands were calculated by subtracting the fast fluorescence value recorded in plants control from those recorded in plants under stress and complementary light spectrum. The results showed that under without and salinity-stressed conditions, all supplementary light treatments caused a decrease in L and K bands compared to ambient light treatment. The blue supplementary light treatment had the greatest impact on reducing the L and K bands under normal conditions (Fig. 7A, B). Incontrast white supplementary light treatment had the greatest impact under salt stress treatment (Fig. 7C, D). In alkalinity stress conditions, the red and white supplementary light treatments resulted in lower L and K bands than ambient light treatment. In addition, the complementary blue and blue/red light treatments increased these bands compared to ambient light treatment (Fig. 7E, F). In salinity/alkalinity stress conditions, the blue/red supplemental light treatment decreased the L band compared to other light treatments, and all supplemental light treatments increased the K band compared to ambient light treatment (Fig. 7G, H).

A comprehensive analysis of different light spectra and stress-induced changes in the OJIP curve was conducted by plotting the differential curves separately for the H and G bands along the O-P curve. These curves were generated by subtracting the fluorescence values (normalized between J-I and I-P) observed in control plants from the values recorded under different light spectra and stress conditions.

**Fig. 8:**
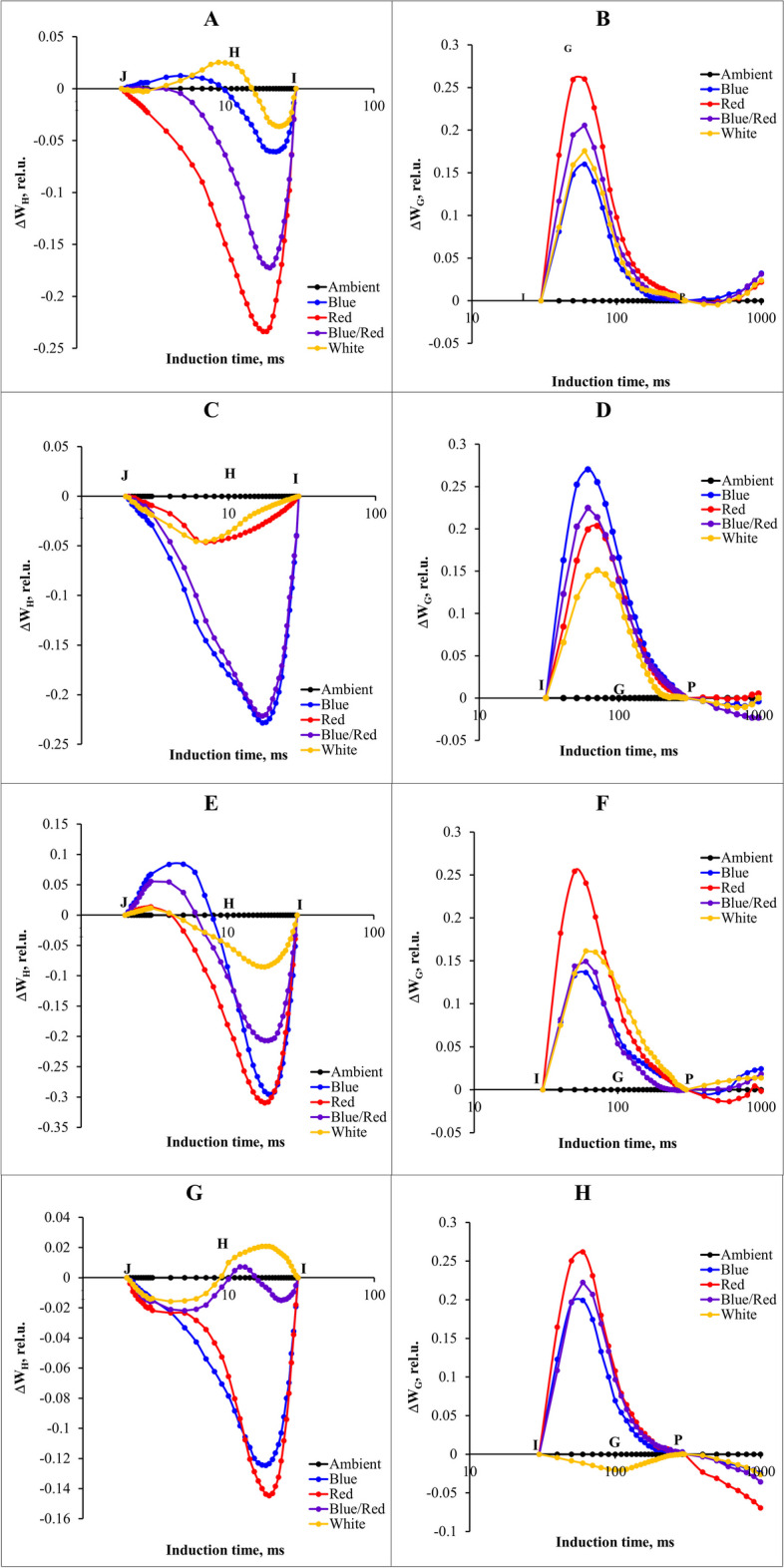
Differential curves of ΔW H and ΔW G of chlorophyll a fluorescence in strawberry cv.Sabrina under various stress conditions. **A**, **B** control; **C**, **D** salinity; **E**, **F** alkalinity; **G**, **H** salinity/alkalinity stress. These stressors were applied across different light spectra, including blue, red, blue/red, white, and ambient light. The biophysical parameters were evaluated using “PEA Plus” software version 1.12, accessible at http://www.hansatech-instruments.com. Graphs were drawn using Microsoft Excel version 2016, which can be found at https://www.microsoft.com

The study findings revealed that under control conditions, red supplementary light led to a significant decrease in the H band. Both blue and white supplementary lights increased this band, although their effects on the G band varied; blue and white supplementary lights exhibited both increases and decreases in the G band, while they mitigated the effects of stress (Fig. 8A, B).

In salinity stress conditions, blue and blue/red complementary lights had the most positive effect on the H band. Conversely, white light induced a significant decrease in the G band and had a positive effect on reducing stress effects (Fig. 8C, D). Moreover, blue, red, and blue/red complementary lights followed similar trends and reduced the H band compared to the ambient light treatment. Additionally, red light increased the G band, whereas other light treatments decreased this band and mitigated stress effects (Fig. 8E, F). Plants subjected to salinity/alkalinity stress and grown under red and blue light exhibited lower H bands, with red light causing a significant increase in the G band (Fig. 8G, H).

### JIP-test parameters

**Fig. 9:**
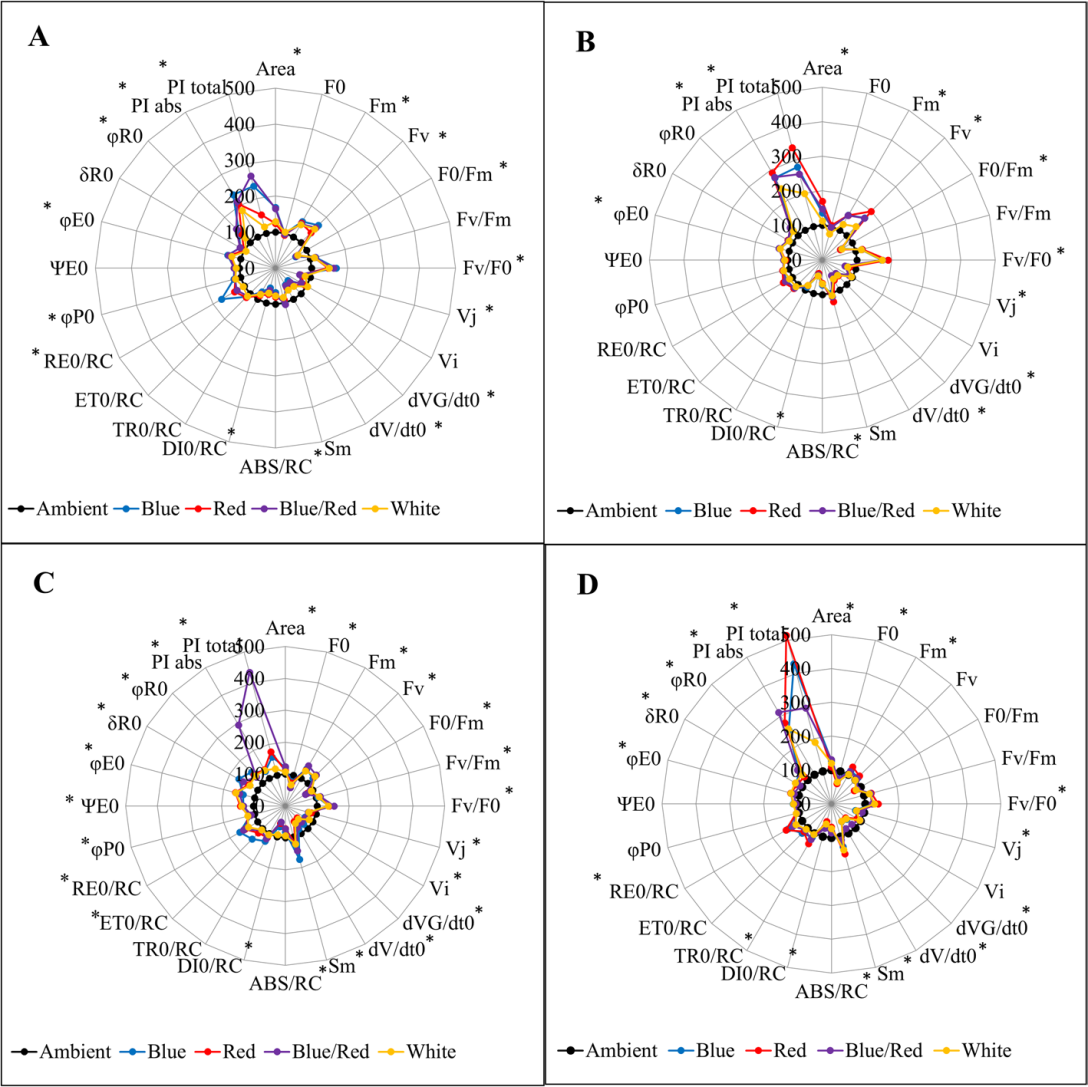
JIP-test parameters, normalized and presented on radar plots, illustrate **A** control, **B** salinity stress, **C** alkalinity stress, and **D** combined salinity and alkalinity stress under different light spectra (blue, red, blue/red, white, and ambient light). Within parameter marked by an asterisk there are significant differences between control and others treatments. The measurement of biophysical parameters was conducted using PEA Plus software version 1.12, available at http://www.hansatech-instruments.com, and the graphical representations were drawn using Microsoft Excel software version 2016, accessible at https://www.microsoft.com

The study conducted on OJIP points produced some interesting results. The points were converted to biophysical parameters such as specific energy fluxes, performance indices, and quantum yields for primary photochemistry, slopes, and integrals. The research revealed that supplementary light treatments induced significant changes in these parameters, especially in different stress conditions. In control conditions, complementary light treatments, particularly blue and blue/red light, caused an increase in P point and variable fluorescence. Plants grown under these light treatments had higher efficiency of the water-splitting complex on the donor side of PSII, quantum yield (φ_P0_, φ_E0_, φ_R0_, Ψ_E0_, δ_R0_), and yield index (PI_ABS_ and PI_total_). However, the V_J_, V_I_, ABS/RC, DI_0_/RC, and TR_0_/RC parameters decreased in comparison to other light treatments (Fig. 9A). In salinity stress conditions, red supplementary light had a significant positive effect on increasing the performance index parameters (PI_ABS_ and PI_total_). Furthermore, under this condition, red supplementary light also caused an increase in variable fluorescence, F_v_/F_0_, and RE_0_/RC (Fig. 9B). In alkalinity stress conditions, plants that were grown under the influence of blue/red supplemental light had the highest P point, variable fluorescence, F_v_/F_0_, and hence, the highest yield index (PI_ABS_ and PI_total_) (Fig. 9C). In salinity/alkalinity stress conditions, plants grown under red, blue, and combination light treatments had the highest yield index (PI_ABS_ and PI_total_) compared to other light treatments. In such conditions, red light had the most significant effect on P point, variable fluorescence, F_v_/F_0_, and RE_0_/RC (Fig. 9D).

### Correlation analysis

**Fig. 10:**
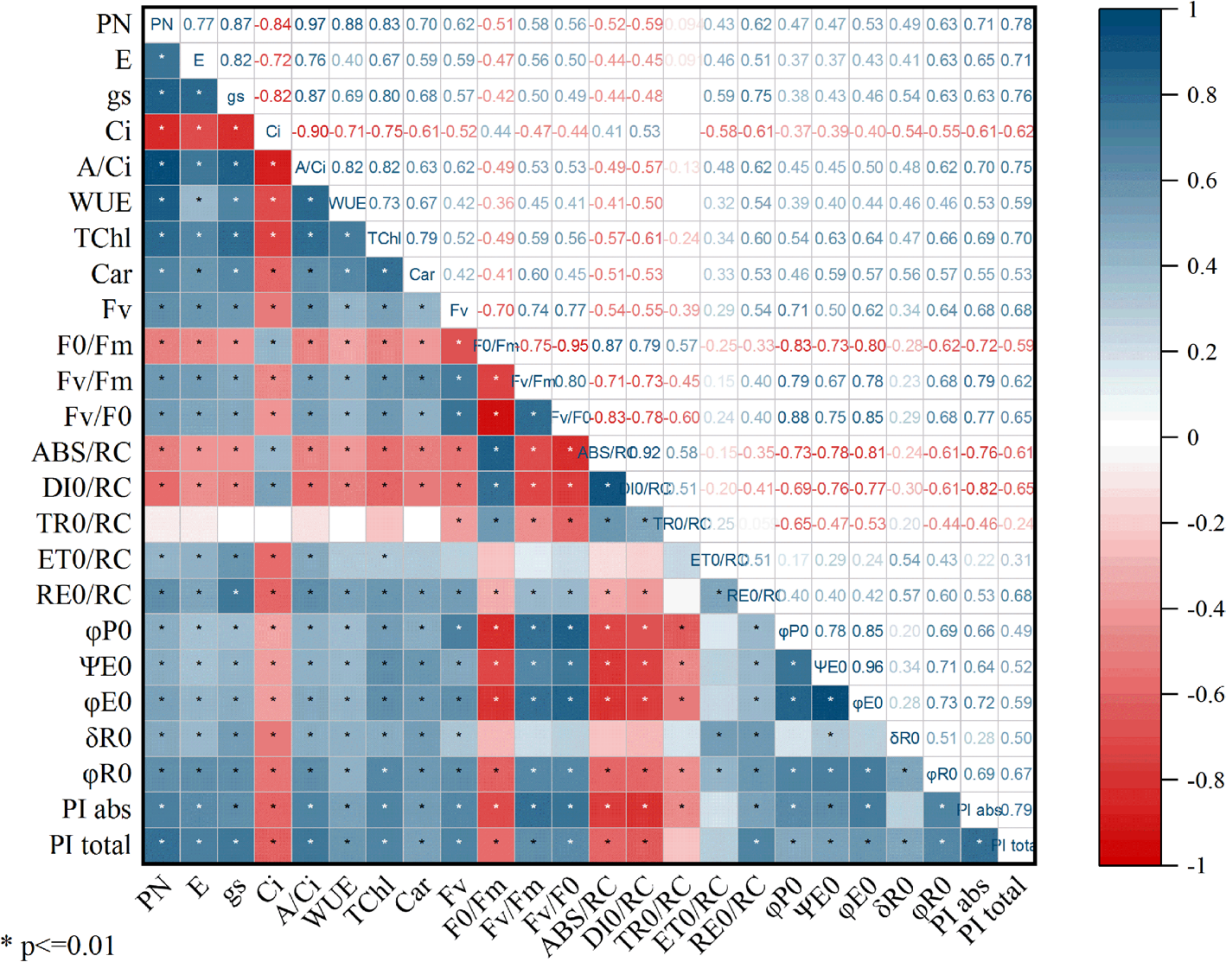
Correlation plot between plant gas exchange, photosynthetic pigments, and JIP test parameters under the influence of five levels of complementary light spectrum and four stress levels. The circles on the plot vary in size and color intensity based on the Pearson correlation coefficient at *p* < 0.01. Positive correlations are represented by blue circles, while negative correlations are denoted by red circles. The Pearson correlation coefficient, ranging from -1 to +1, is indicated on the correlation scale for variables on the vertical and horizontal axes. Values marked with *are statistically different at *p* < 0.01. The correlation diagram was created using Origin Pro software version 2021, accessible at https://www.originlab.com/2021

In Fig. 10, the correlation plot displays the correlation between plant gas exchange, photosynthetic pigments, and JIP test parameters. The findings suggest that *C*_*i*_, F_0_/F_m_, ABS/RC, and DI_0_/RC had negative correlations with other plant gas exchange and JIP test parameters. The most significant negative correlation was found between *C*_*i*_ parameters with *A/C*_*i*_ and F_V_/F_0_ with F_0_/F_m_. The *P*_*N*_ parameter had a strong positive correlation with *E*, *g*_*s*_, *A/C*_*i*_, *WUE*, and TChl parameters. On the other hand, the F_0_/F_m_ parameter had a negative correlation with the quantum yield parameters, especially φ_P0_, Ψ_E0_, and φ_E0_, as well as with F_V_/F_0_. Furthermore, the F_0_/F_m_ parameter had a significant positive correlation with ABS/RC and DI_0_/RC parameters. Finally, total chlorophyll and carotenoids showed a positive correlation with quantum yield and yield index parameters.

### Principal component analysis

The first step involved standardizing the data to achieve a zero mean and unit variance. Following this, Principal Component Analysis (PCA) was conducted to capture the variations of 15 parameters across five light spectrum treatments and two distinct stress levels. The magnitude of each vector in the PCA signifies the impact of each parameter, with direction determined by the values of PCA1 and PCA2. Additionally, another PCA was performed to encapsulate variations of 24 parameters across five light spectrum levels under various stress conditions: (A) no stress, (B) salinity stress, (C) alkalinity stress, and (D) combined salinity/alkalinity stress.

**Fig. 11:**
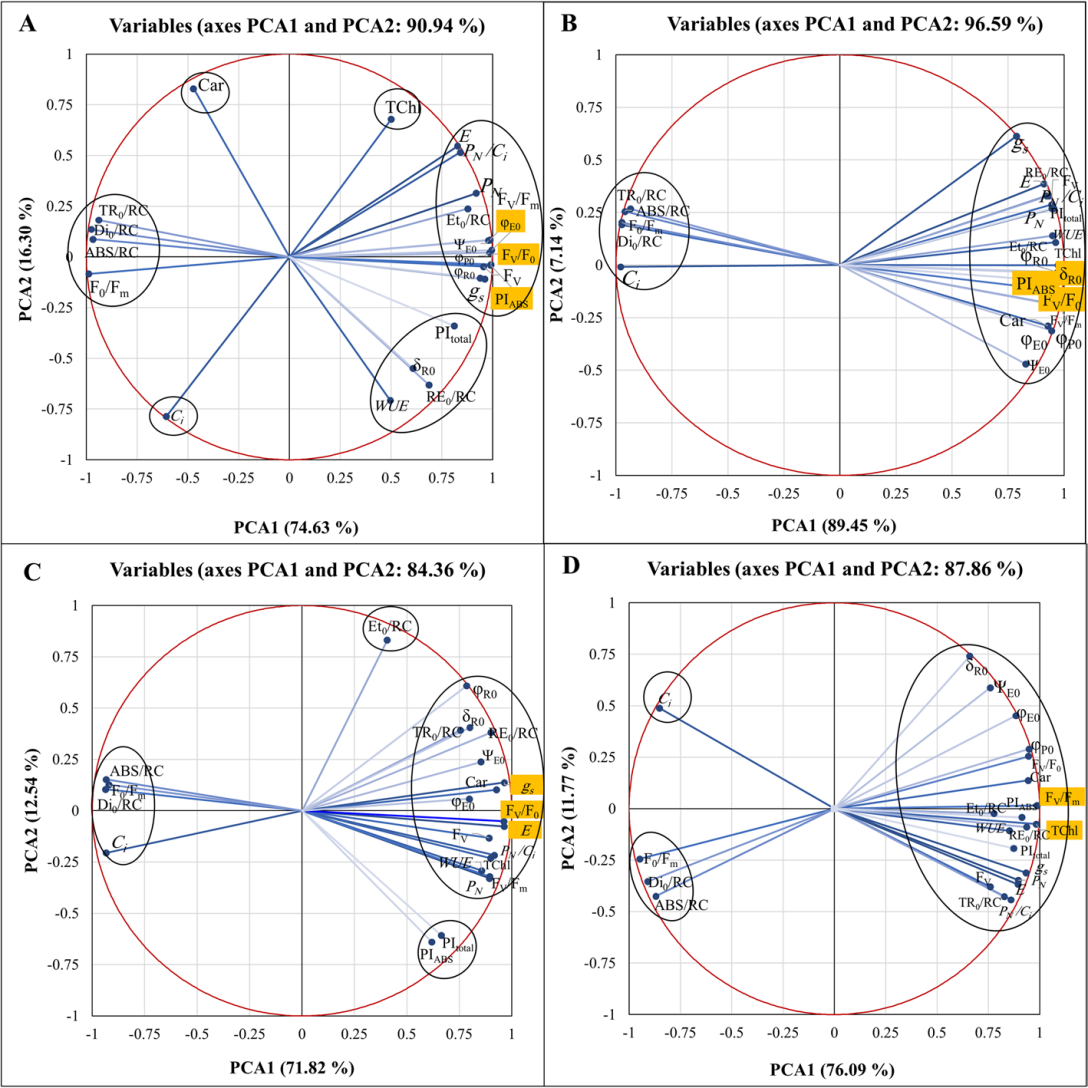
Biplot representation of principal components for leaf gas exchange, photosynthetic pigments, and JIP test parameters, reflecting variations across five levels of light spectra under (**A**) control, (**B**) salinity stress, (**C**) alkalinity stress, and (**D**) combined salinity and alkalinity stress

 In the control condition, PCA1 and PCA2 accounted for 90.94% of the total variance observed in the five levls of light spectrum treatments (as shown in Fig. 11A). The corresponding values for salinity, alkalinity, and combined salinity/alkalinity stress treatments were 96.59%, 84.36%, and 87.86%, respectively (illustrated in Fig. 11B, C, D). Irrespective of the direction of their effects, parameters P12 (F_V_/F_0_), P20 (φ_E0_), and P23 (PI_ABS_) made the most significant contributions to the first principal component (PCA1) in the control treatment with the five light spectrum levels. In the salinity stress treatment, the parameters P12, P21, and P23; in the alkalinity stress treatment, P2, P3, and P12; and the combined salinity/alkalinity stress treatment, P7 and P11, were the most influential in driving the changes observed with the different light spectrum levels.

## Discussion

A decline in chlorophyll concentration over the growing season can significantly impact photosynthesis, plant growth, development, and stress tolerance. Under conditions of salinity stress, there is an increase in the activity of the enzyme chlorophyllase, leading to chlorophyll degradation. Consequently, as the stress level rises, there is a more pronounced reduction in the plant’s chlorophyll content [4, 29]. The decrease in chlorophyll content, induced by heightened chlorophyllase enzyme activity under salinity and alkalinity stress, is associated with the accumulation of specific ions. Sodium and chlorine ions accumulate under salinity stress, while sodium and bicarbonate ions build up under alkalinity stress. These ions impede the uptake of essential cations like magnesium, iron, potassium, and calcium. As these cations are crucial for chlorophyll structure and synthesis, their reduced absorption significantly impacts chlorophyll content [30]. Additionally, the decline in chlorophyll concentration may also result from magnesium precipitation at high pH levels, which hinders chlorophyll synthesis [31]. In salinity and alkalinity stress conditions, the disruption caused by an excess of certain ions, such as sodium, can inhibit proteinase activity. This imbalance adversely affects chlorophyll concentration, leading to a reduced photosynthesis rate in plants [32]. Iron deficiency can further contribute to a decrease in chlorophyll concentration. Iron, as a vital element, plays a crucial role in various metabolic and enzymatic processes, including chlorophyll biosynthesis, electron transport, photosynthesis, and nitrogen fixation [4].

While salinity stress generally leads to a decrease in chlorophyll content, the extent of this decrease varies according to plant species tolerance to salinity. As a result, chlorophyll levels have been proposed as a biochemical marker to evaluate plant tolerance to salinity [33]. Throughout plant growth and development, high pH levels induced by stress can trigger chemical reactions that convert iron in the nutrient solution into an insoluble solid. This alteration renders the iron inaccessible to plant roots, even when an adequate iron supply is available [34]. Salinity stress disrupts photosynthesis by causing stomatal closure, impairing photochemical reactions, and hindering carbon absorption. The closing of the stomata acts as the primary defense response of the plant to salinity and alkalinity stress [35]. CO_2_ entry into the leaf is reduced under stress conditions, which disturbs the photosynthetic electron transfer chain [36]. Our research indicates that salinity, alkalinity, and their combined effects contribute to reduced electron absorption and trapping within the electron transport chain. This leads to an elevated ABS/RC and TR_0_/RC across all stress conditions, primarily attributable to the loss of active reaction centers [37]. The water-splitting complex (OEC) is a crucial and sensitive part of the electron transport chain of the photosynthetic apparatus [38]. Its efficiency is reduced due to the disruption of the electron transport process, which is caused by the dissociation of light-receiving complexes (LHCII). Research on tomato seedlings has demonstrated that photosystem II electron transfer is affected by salinity/alkalinity stress [39]. Salt stress has been shown to reduce the activity of photosystem II by affecting the manganese cluster and the activity of photosystem I [40], causing the production of reactive oxygen species (ROS) and the destruction of the D_1_ protein [41] by separating plastocyanin from cytochrome C_553_. This revealed that the electron transport chain of the photosynthetic apparatus and plant gas exchange parameters are vulnerable under stress conditions [42]. Our research shows that exposure to salinity, alkalinity, and their combined stress negatively affects photosystem II, leading to increased energy loss (Fig. 9). As seen in Fig. 7, this is accompanied by an increase in the initial fluorescence level (F_0_), indicating the loss of energy as heat and a decrease in the initial slope associated with the decrease of the K band in the chlorophyll fluorescence induction curve. Stress conditions cause a decrease in F_m_, indicating a disruption in the flow of electrons in photosystem II due to non-photochemical quenching, inactivation of photosystem II reaction centers, and degradation of D_1_ protein [43]. The results of our study indicate that exposure to salinity and alkalinity stress can lead to a decrease in the flow of electrons in the receptor side of photosystem (I) This decrease can be attributed to the inactivation of ferredoxin NADP reductase, resulting in a decrease in the values of φ_R0_ and RE_0_/ABS [44].

Carotenoids serve as vital precursors in signaling pathways during plant growth under abiotic stress, offering protection to membranes against oxidative damage [45]. Chloroplasts predominantly absorb blue and red light for photosynthesis [46], with photosynthetic pigments playing a pivotal role in light energy absorption and transfer during photosynthesis [47]. Previous studies have highlighted that the expression of key genes encoding enzymes related to chlorophyll and carotenoid pigments increases with both blue and red-light treatments. These treatments consequently lead to heightened pigment accumulation and light absorption, thereby enhancing photosynthesis [48]. Huang et al. demonstrated that combining blue and red light can elevate chlorophyll concentration [49]. In our study, we observed that blue/red and red treatments yielded higher chlorophyll levels compared to other light treatments (Fig. 3).

Manipulating the light spectrum using LEDs has been shown to increase zeaxanthin concentration, facilitating stomatal opening and thereby enhancing plant tolerance to stress conditions [50]. Hernandez and Kubota have suggested that improving the quality of light received by plants, particularly through blue and red lights, can result in optimal growth and increased tolerance to high-stress conditions. This enhancement is attributed to LED lights’ capacity to elevate the concentration of antioxidant compounds in plants [51].The capacity of plants to assimilate nutrients and generate biomass predominantly hinges on their photosynthetic rate. Studies indicate that treatments involving red and blue/red light can markedly enhance net photosynthetic rates. This is primarily attributed to the preferential absorption of red light by Photosystem II, rendering such treatments more efficacious. Prolonged exposure to red light can disrupt the balance of excitation rates between photosystems, leading to an increased stimulation rate of both Photosystems I and II [52]. Additionally, other research has demonstrated that exposure to red light enhances the efficiency of immediate carboxylation [53].

Water use efficiency (*WUE*) denotes the amount of carbon absorbed per unit of water consumed by the plant. Simultaneously, internal Water Use Efficiency (*WUEi*) is defined as the ratio of net carbon dioxide uptake to stomatal conductance. Supplementary lighting, particularly in the red and blue/red light spectrum, has exhibited a notable effect on various parameters under any stress condition. Specifically, blue light projected against a red background has proven effective in augmenting photosynthesis [54, 55]. Furthermore, combining red and blue light can significantly enhance the activity of enzymes associated with photosynthesis formation and augmentation [56].

It is widely recognized that red light induces stomatal opening in isolated epidermis, with the response relying on the chloroplasts of the guard cells [57]. Stomata on a leaf play a pivotal role in facilitating air and water exchange with the surrounding environment. The various spectra of light received by plants influence the dynamic behavior of leaf stomata, thereby affecting the rate of air and water exchange, ultimately impacting the plant’s photosynthetic efficiency [58]. The response of stomata to red and blue light is delineated into two components: a photosynthesis-independent component and a photosynthesis-dependent component [59, 60]. The former can promptly trigger stomatal opening through blue light exposure [61], while the latter is mediated by red light via photosynthesis [62]. This could elucidate the impact of red and blue/red light on the electron transport chain. The parameter " *g*_*s*_” denotes the plant’s capacity to assimilate carbon dioxide through stomata, consequently influencing the photosynthetic rate [63]. Blue light promotes stomatal development [64, 65], yet blue/red light exerts a more pronounced effect on stomatal opening [63]. Under stress conditions, the influence of red and blue/red light on stomatal aperture regulation can aid in maintaining a steady state. Blue light exhibits a greater influence on this parameter under non-stress conditions. In comparison to monochromatic light, the combination of blue/red light results in higher “*g*_*s*_” likely because treatment with a high proportion of red light can enhance the expression of stomatal growth genes such as ZmSPCH and ZmMUTE [66]. The coordinated regulation of all constituents of the photosynthetic apparatus ensures the internal equilibrium between the efficiency of photosynthetic reactions and the efficiency of reactions involved in CO_2_ assimilation [67]. Plants predominantly absorb blue and red light (approximately 90%). Besides direct absorption by the photosystem, blue light can indirectly impact stomatal aperture, which may occur independently of photosynthetic activity (CO_2_ reduction in the leaf), thereby increasing transpiration without significantly affecting photosynthesis [68]. Stomatal opening induced by red light results from both the response of stomatal guard cells to a decrease in intercellular CO_2_ concentration and the direct response of chloroplast guard cells to red light [69]. Both blue and red light are crucial for chlorophyll synthesis [70]. Stressful conditions exert a deleterious effect on the photosynthetic apparatus, disrupting the electron transfer process [71].

Plants grown with supplemental light showed a significant increase in Ψ_E0_ and φ_E0_ parameters that could lead to better functioning of photosynthetic apparatus and then enhancing plant resilience to salinity and alkalinity. The stress condition led to an increase in the electron transport current at each reaction center and the excitation probability that moves the electron in the post Q_A_^-^ electron transport system. The results demonstrate that the additional light has enhanced the plants’ capacity to transport more electrons from the photons absorbed in photosystem (II) This improvement means that the energy level in the reaction centers is positively regulated, and the electrons can move beyond Q_A_^-^. As shown in Fig. 11, the parameters of quantum performance and performance index and efficiency of water-splitting complexes had the largest contribution to the total changes of five optical levels under all stress conditions. Stress conditions lead to a reduction in CO_2_ availability due to increased penetration resistance in stomatal and mesophyll cells [36]. In such conditions, the photosynthetic capacity of plants can be influenced by different wavelengths of supplementary light. As depicted in Fig. 10, the correlation between gas exchange and chlorophyll fluorescence parameters illustrates this phenomenon. The plant strives to maintain a balance between carbon regeneration and electron transfer to augment its photosynthetic capacity. These processes underscore a complex relationship between the wavelengths received by plants, the impact of environmental stressors, and photosynthetic responses. It is feasible to optimize the activity of the photosynthetic apparatus by adjusting light spectrum conditions under stress, thereby affecting the production of light energy and carboxylation demand. When stress reduces the energy demand, excess energy can lead to damage or a reduction in the photo phase capacity in photosystems [72]. Modifying the wavelengths of light received by plants enables us to influence stomatal behavior, energy supply, and demand. Our findings revealed that white light also positively impacted the photosynthetic apparatus of strawberry plants when compared to ambient light conditions. The white spectrum encompasses all visible light colors from blue to red. Different light spectra can evoke distinct responses in the photosynthetic apparatus of plants, with each color within the white spectrum possessing specific wavelengths and energy levels.

When plants are exposed to white light, photosynthetic pigments can absorb various wavelengths of light within the spectrum. Each chlorophyll molecule exhibits a characteristic absorption spectrum, with chlorophyll a predominantly absorbing light in the blue and red regions, while chlorophyll b shows absorption peaks in the blue and yellow-green regions of the spectrum. The white spectrum enables the absorption of light across a broad range of wavelengths by these pigments, facilitating optimal energy capture for photosynthesis. Furthermore, the white spectrum has been demonstrated to promote overall plant growth.

The presence of all colors within the white spectrum ensures that plants receive a balanced array of different wavelengths, crucial for various physiological processes. Additionally, the white spectrum provides the requisite energy for synthesizing chlorophyll, enzymes, and other components involved in photosynthesis. This could explain the positive impact of white light on the levels of chlorophyll and carotenoids compared to ambient light, particularly in stress conditions, as observed in the results of this experiment. The availability of diverse wavelengths in the white spectrum allows plants to efficiently absorb light energy and enhance photosynthesis.

Understanding the effect of supplemental light on the electron transport chain in strawberry plants provides insights into how light influences the photosynthetic machinery at the molecular level. Supplemental light can enhance the rate of electron flow through the electron transport chain. Supplementary light increases the availability of electrons from water molecules and the rate of electron flow through the electron transport chain, thereby helping to increase the proton gradient and optimize ATP production [73]. In addition to ATP, Supplemental light can stimulate the synthesis of NADPH by promoting efficient electron transport through the chain [74]. Supplemental light helps maintain a balance in redox reactions, preventing the accumulation of excess electrons and minimizing the potential for oxidative stress. This balance is crucial for the overall health and productivity of strawberry plants [75]. Understanding the intricate relationship between supplemental light and the electron transport chain in strawberry plants requires a holistic approach that considers light intensity, duration, and quality [76].

## Conclusions

When plants encounter environmental stress, they employ various adaptive strategies influenced by the quality of light they receive during photosynthesis and gas exchange. In such circumstances, red and blue/red light spectrums can significantly improve the efficiency of the photosynthetic apparatus. This improvement stems from their positive impact on the electron transport chain and the enhancement of plant gas exchange parameters, surpassing the effects of other light treatments. By examining and comprehending how different complementary light spectra affect plants in diverse growth conditions, it becomes feasible to manipulate the light spectrum received by plants, thereby enhancing their stress tolerance. Our findings demonstrate that enhancing the quality of light received enables plants to augment their photosynthetic activity, absorb maximum light energy, and influence their response to environmental stresses. These results underscore the pivotal role of complementary light spectrums in energy absorption, regulation of growth and development, and physiological functions of plants. Complementary light spectrum can yield multiple functional consequences on plants, including effects on growth and development, germination and flowering, regulation of photosynthesis, morphology, and response to environmental stresses. As technology and horticultural research progress, growers can capitalize on optimized lighting strategies to elevate the photosynthetic efficiency of strawberry plants and enhance overall crop productivity.

### Supplementary Information

**Supplementary Material 1.**

## Data Availability

The authors declare that the data supporting the findings of this study are available within the paper and its Supplementary Information files. Should any raw data files be needed in another format they are available from the corresponding author upon reasonable request. Source data are provided with this paper.
