# Supplementary material for: Improving strawberry plant resilience to salinity and alkalinity through the use of diverse spectra of supplemental lighting
Source: BMC Plant Biol. 2024 Apr 8;24:252. doi: 10.1186/s12870-024-04984-y (PMC11000407; doi:10.1186/s12870-024-04984-y)
Supplement: Supplementary file 1 — Supplementary Material 1. [file 12870_2024_4984_MOESM1_ESM.docx]

| Basic parameters derived from the extracted data | | |
| --- | --- | --- |
| F_0_ | Minimal fluorescence, when all PSII RCs are open | F_0_ = F20μs |
| F_m_ | Maximal fluorescence, when all PSII RCs are closed | F_m_ (= FP) |
| F_v_ | Maximal variable fluorescence | F_v_ = F_m_ – F_0_ |
| Area | the area between fluorescence curve and F_M_ or Area above the fluorescence curve |  |
| Normalized data | | |
| F_v_/F_m_ | The maximum quantum yield of PSII | F_v_/F_m_= φP_0_ = TR_0_/ABS = [1− (F_0_/F_m_) |
| F_0_/F_m_ | A parameter related to changes in heat dissipation in the photosystem II antenna | 1 – F_v_/F_m_ |
| F_v_/F_0_ | The efficiency of the water-splitting complex on the donor side of PSII. | (F_m_ – F_0_)/F_0_, 1/ (1 – F_v_/F_m_) −1, 1/(F_0_/F_m_) −1 |
| V_j_ | Relative variable fluorescence at the J-step (t=2ms) | (F_2ms_ - F_0_) / (FM - F_0_) |
| V_i_ | Relative variable fluorescence at time 30 ms (I-step) after the start of the actinic light pulse | V_i_ = (F_30ms_ – F_0_)/ (F_m_ –F_0_) |
| Specific energy fluxes (per active PSII reaction center) | | |
| ABS/RC | Absorption flux (of antenna Chls) per RC (also a measure of PSII apparent antenna size) | M_0_ (1/V_J_) (1/φP_o_) |
| DI_0_/RC | Dissipated energy flux per RC at t = 0 | ABS/RC -TR_0_/RC |
| TR_0_/ RC | Maximal trapping rate of PSII | M_0_=Vj |
| ET_0_/ RC | Electron transport in active RC | M_0_. (1 / V_J_). ψ_0_ |
| RE_0_/ RC | Electron flux leading to the reduction of the PSI end acceptor | M_0_ (1/V_J_) (1 - V_I_) |
| performance indexes | | |
| PI_(abs)_ | The performance index for energy conservation from excitation to the reduction of intersystem electron acceptors | PI_ABS_ = (γRC/1- γRC) (φP_0_/1 - φP_0_) (ψE_o_/1- ψE_o_) |
| PI_(total)_ | The performance index for energy conservation from excitation to the reduction of PSI end acceptors | PI_total_ = PI_ABS_. δR_0_/ (1 - δR_0_) |
| The quantum yield for primary photochemistry | | |
| φ_P0_ | Maximum quantum yield of primary PSII photochemistry (when all RCs are open, V=0) | φP_o_ = TR_o_ /J_ABS_ = 1 _ F_o_/F_M_ |
| Ψ_E0_ | Efficiency/probability that an electron moves further than QA- | ET_0_/TR_0_ = 1 - V_J_ |
| φ_E0_ | Quantum yield for electron transport (ET) | ET_0_/ABS = (F_v_/F_m_) × (1 - V_J_) |
| δ_R0_ | The efficiency with which an electron from the intersystem electron carriers moves to reduce end electron acceptors at the PSI acceptor side (RE) | RE_0_/ET_0_ = (1 - V_I_)/ (1 - V_J_) |
| φ_R0_ | It expresses the probability that an absorbed photon leads to a reduction of the PSI end acceptor | φP_o_+ΨE_0_+ δR_0_ = RE_0_/ABS |
| Slopes and integrals | | |
| dVG/dt_0_ | Express the excitation energy transfer between the reaction centers |  |
| dV/dt_0_ | Express the rate of the reaction centers closure |  |
| S_m_ | Normalized area; is related to the number of electron carriers per electron transport chain | S_m_ = Area/ (F_m_ − F_0_) = Area/F_V_ |
| Leaf Gas Exchange | | |
| *A* | CO_2_ Assimilation Rate (μmol (CO_2_) mol^-1^) |  |
| *E* | Transpiration rate (mol m^-2^. s^-1^) |  |
| *G_s_* | stomatal conductance (mol (H_2_O) m^-2^. s ^-1^) |  |
| *C_i_* | Sub-stomatal CO_2_ concentration (μmol mol^-1^) |  |
| *A/C_i_* | instantaneous carboxylation efficiency |  |
| *WUE* | water-use efficiency (μmol (CO_2_) mol H_2_O^−1^) | *A/E* |
| *WUEi* | intrinsic water use efficiency | *A/ Gs* |

The description of fluorescence parameters (Strasser et al. 2010) and Leaf Gas Exchange parameters
